# Discrepancy-Guided Complementary Fusion for Unsupervised Multimodal Anomaly Detection

**DOI:** 10.3390/s26123757

**Published:** 2026-06-12

**Authors:** Taehui Lee, Seyoung Jeong, Sang Jun Lee

**Affiliations:** Division of Electronic Engineering, Jeonbuk National University, 567 Baekje-daero, Deokjin-gu, Jeonju 54896, Republic of Korea; aqaxz123@jbnu.ac.kr (T.L.); seyoung@jbnu.ac.kr (S.J.)

**Keywords:** industrial anomaly detection, multimodal anomaly detection, multi-sensor fusion, feature-level fusion, feature extraction, unsupervised learning

## Abstract

In industrial inspection, subtle defects often appear as local variations in appearance or geometry, making reliable anomaly detection challenging. A single sensing modality can miss important defect cues, while multimodal inspection combines appearance and geometric information to represent industrial objects more comprehensively. Many existing multimodal anomaly detection methods adopt early fusion strategies that integrate features at an early stage of the network. Such early integration can dilute modality-specific anomaly responses and cause anomaly smoothing, leading to degraded detection and localization performance. To address these challenges, we propose a reconstruction-based unsupervised multimodal anomaly detection framework integrating Discrepancy-Guided Complementary Fusion (DGCF) and Noise to Feature (N2F). Specifically, DGCF reduces anomaly smoothing by exploiting cross-modal discrepancies to extract complementary information, rather than directly summing or concatenating features from different modalities. Furthermore, N2F injects Gaussian noise into the feature space to regularize feature reconstruction and encourage the decoder to learn robust normal representations. Experimental results on the MVTec 3D-AD and Eyecandies datasets demonstrate the effectiveness of the proposed method. The proposed method achieves 97.3% I-AUROC, 99.6% P-AUROC, and 97.6% AUPRO on MVTec 3D-AD, and 94.8% I-AUROC, 98.6% P-AUROC, and 93.4% AUPRO on Eyecandies.

## 1. Introduction

In manufacturing environments, anomaly detection has mainly relied on manual visual inspection by human operators. However, such inspection is limited by inconsistent detection performance, which can vary depending on inspection speed and the operator’s experience. Accordingly, artificial intelligence (AI)-based anomaly detection methods have been actively investigated. Nevertheless, supervised anomaly detection remains challenging in real industrial environments because abnormal samples rarely occur and anomalous regions exhibit large variations in size, location, and shape [1]. To overcome these limitations and improve the accuracy and reliability of visual inspection systems, unsupervised anomaly detection methods that learn only from normal data have been widely studied [2,3,4].

As manufacturing processes become more advanced and inspection targets become more diverse, industrial anomalies also appear in increasingly complex forms. Anomalies may appear as appearance defects, such as color changes or contamination on object surfaces, or as geometric defects, such as shape deformation or depth differences. As the types of anomalies become more diverse, it is difficult to capture all anomaly-related information using a single modality, such as RGB images. For this reason, multimodal anomaly detection methods that jointly utilize information from different modalities, such as RGB, Depth, and Normal, have attracted increasing attention to improve anomaly detection performance.

In multimodal anomaly detection, the way information from different modalities is fused has a significant impact on detection performance. Existing multimodal anomaly detection methods have widely adopted direct feature fusion strategies that combine features from different modalities, such as RGB, Depth, and Normal [5]. Although these direct fusion strategies can utilize information from multiple modalities simultaneously, they often combine features without sufficiently considering the modality-specific characteristics and anomaly cues. In particular, when an anomaly is clearly observed in one modality but appears similar to normal patterns in another modality, simple feature fusion may weaken the anomaly signal rather than enhance it. This indiscriminate fusion of cross-modal information can lead to an anomaly smoothing problem, in which important anomaly cues are diluted during the fusion process. Therefore, multimodal anomaly detection requires a fusion strategy that not only combines information from different modalities but also preserves modality-specific anomaly cues while effectively exploiting complementary information.

In addition to this fusion-related limitation, reconstruction-based anomaly detection methods face another challenge in the reconstruction process itself. Feature reconstruction-based anomaly detection methods train a decoder to reconstruct feature representations of normal data and use the difference between the input features and the reconstructed features as an anomaly score, based on the assumption that abnormal regions cannot be sufficiently reconstructed because they differ from normal patterns [6,7]. However, as the representation capacity of the decoder increases, abnormal regions contained in the input features may also be reconstructed well. In this case, the difference between the input and reconstructed features decreases, which weakens the anomaly score and consequently degrades the final detection performance [8,9].

To address these issues, we propose a reconstruction-based unsupervised multimodal anomaly detection framework that consists of two key modules: DGCF and N2F. DGCF mitigates anomaly smoothing by exploiting cross-modal discrepancy information to extract complementary features, rather than directly summing or concatenating features from different modalities. By selectively emphasizing modality-discrepant information, DGCF preserves anomaly cues that may be prominent in one modality but weakened in another. N2F further improves reconstruction learning by injecting Gaussian noise into the feature space, which discourages the decoder from simply copying input features and encourages it to learn robust normal representations. Through these two modules, the proposed framework effectively addresses both fusion-related anomaly smoothing and reconstruction-related over-reconstruction. Experimental results on the MVTec 3D-AD and Eyecandies datasets demonstrate that the proposed method achieves consistent improvements in both quantitative metrics and qualitative results.

In summary, the main contributions of this paper are as follows:

- We propose a reconstruction-based unsupervised multimodal anomaly detection framework that alleviates both anomaly smoothing in feature fusion and over-reconstruction in feature reconstruction.
- We introduce a DGCF module that exploits cross-modal discrepancy information to selectively enhance complementary features while preserving modality-specific anomaly cues.
- We propose an N2F module that injects Gaussian noise into the feature space to discourage simple feature copying by the decoder and improve the robustness of normal feature reconstruction.

The remainder of this paper is organized as follows. Section 2 reviews related work on industrial artificial intelligence, feature embedding-based anomaly detection, reconstruction-based anomaly detection, and multimodal anomaly detection. Section 3 presents the proposed reconstruction-based unsupervised multimodal anomaly detection framework, including the DGCF module, the N2F module, and the anomaly scoring strategy. Section 4 describes the experimental setup, datasets, evaluation metrics, comparative results, qualitative analysis, and ablation studies. Finally, Section 5 concludes the paper.

## 2. Related Work

### 2.1. Industrial Artificial Intelligence

In recent years, AI has been increasingly applied in industrial environments to improve production efficiency and product quality. AI-based techniques have been applied to various manufacturing tasks, including equipment maintenance, logistics optimization, and quality inspection, and are increasingly used to enhance the efficiency and stability of manufacturing processes. In particular, traditional maintenance practices have mainly relied on reactive responses after equipment failure or periodic replacement despite normal operation. To overcome these limitations, Dida et al. [10] proposed a method that utilizes time-series sensor data to predict equipment failures in advance and estimate appropriate maintenance timing. In logistics systems, AI-based approaches have also been used to optimize delivery routes by considering customer locations and demand [11], and to reduce inventory management costs through product demand forecasting [12].

Among these applications, AI-based quality inspection is particularly important in manufacturing processes because it enables the automatic detection of visual defects on products. However, industrial anomaly detection is challenging because most collected samples are normal, while abnormal samples occur rarely and appear in diverse forms. These characteristics make it difficult to train supervised learning-based models that require sufficient abnormal examples. Therefore, unsupervised anomaly detection methods, which learn normal patterns from normal data only and identify deviations during inference, have been widely investigated for industrial inspection tasks.

### 2.2. Feature Embedding-Based Anomaly Detection

Feature embedding-based anomaly detection methods aim to detect anomalies by modeling the feature distribution of normal data and measuring the degree to which test features deviate from this distribution. These approaches commonly employ pretrained neural networks as feature extractors and use patch-level feature representations to capture local normal patterns. Since robust representations can be obtained without large-scale task-specific training, feature embedding-based methods have shown strong performance in unsupervised industrial anomaly detection.

SPADE [13] detects anomalies by comparing features extracted from normal training images with those extracted from test images. PaDiM [14] models the distribution of patch-level features by estimating the mean and covariance at each spatial location, and computes anomaly scores using the Mahalanobis distance. PatchCore [15] constructs a memory bank with representative normal feature patches and detects anomalies through nearest-neighbor search. By selecting core normal patches rather than storing all extracted features, PatchCore improves computational efficiency while maintaining strong detection performance.

Although feature embedding-based methods are effective for detecting subtle texture changes and local surface defects, their performance largely depends on the quality of pretrained representations and the modeling of normal feature distributions. In addition, methods based on feature matching or nearest-neighbor search often require considerable memory and computational cost during inference. Moreover, since anomalies are detected by measuring deviations in the extracted feature space, capturing complex structural changes or geometric deformations can still be challenging. These limitations indicate that, despite their strong performance, feature embedding-based approaches remain limited in handling certain industrial anomaly detection scenarios.

### 2.3. Reconstruction-Based Anomaly Detection

Reconstruction-based anomaly detection methods have been widely studied based on the assumption that a model trained only on normal data can accurately reconstruct normal patterns but cannot reliably reconstruct abnormal regions. In these methods, a model is trained to reconstruct the input image or feature representation, and anomalies are detected by measuring the difference between the input and its reconstruction during inference. Since anomalous patterns are not observed during training, regions with large reconstruction errors are regarded as abnormal. This intuitive framework has made reconstruction-based approaches one of the representative directions in unsupervised anomaly detection.

However, reconstruction-based methods often suffer from the over-reconstruction problem. When the decoder has excessive reconstruction capability, it may also reconstruct anomalous regions with low reconstruction error. In this case, the difference between the input and reconstructed output becomes less distinguishable, leading to weakened anomaly scores and degraded detection performance. To alleviate this limitation, DDR-ID [16] decomposes input data into normal and abnormal components and performs reconstruction mainly on the normal component. DiffusionAD [17] further addresses the limitations of conventional reconstruction-based approaches by using a diffusion model to generate diverse normal patterns.

Recently, several studies have shifted from image-level reconstruction to feature-level reconstruction. Deng et al. [18] introduced Reverse Distillation (RD), which uses One-Class Bottleneck Embedding (OCBE) to compress encoder feature maps into compact normal representations before passing them to the decoder. The decoder then reconstructs normal feature representations, and anomalies are detected by comparing the reconstructed features with the input encoder features. REASON [19] introduced anomaly-like perturbations into partial feature patches to mitigate over-reconstruction and encourage the model to recover normal feature structures rather than simply copying the input. These studies indicate that controlling the reconstruction process in feature space is important for preserving discriminative anomaly signals. Based on this observation, our method introduces feature-level noise into the reconstruction process to reduce over-reconstruction and improve the robustness of anomaly detection.

### 2.4. Multimodal Anomaly Detection

Most existing anomaly detection methods have been designed based on a single modality, such as RGB images. However, RGB data primarily capture appearance information, including color and texture, and can be sensitive to external environmental factors such as illumination changes. In addition, RGB images may be insufficient for capturing structural defects such as depth variations, dents, or geometric deformations. To overcome these limitations, multimodal anomaly detection methods that utilize different types of sensor data, such as RGB, Depth, and Normal maps, have recently attracted increasing attention.

Multimodal anomaly detection can provide richer information than single-modality approaches by combining appearance and geometric cues. However, effectively integrating heterogeneous modalities remains challenging because each modality has different characteristics and representation spaces. In particular, when features from different modalities are directly summed or concatenated, anomaly cues that are clearly observed in one modality can be weakened by normal-like responses from another modality. This phenomenon can be regarded as anomaly smoothing, where modality-specific anomaly signals become less discriminative during the fusion process. Therefore, the design of the fusion strategy is critical in multimodal anomaly detection.

In response to these challenges, various multimodal fusion strategies have been proposed. CMDR-IAD [20] performs anomaly detection by processing each modality independently while exchanging complementary information at intermediate stages. M3DM [21] points out the limitations of simple feature concatenation and proposes a hybrid fusion strategy that maps features from different modalities into a common feature space and models modality interactions based on patch-level relationships. These studies demonstrate that multimodal anomaly detection has moved beyond simple feature combination toward more sophisticated fusion strategies.

Nevertheless, effectively selecting complementary information between modalities remains an important challenge. Since different modalities may contain inconsistent or modality-specific anomaly cues, fusion methods that do not sufficiently consider cross-modal differences can still weaken discriminative anomaly signals. Therefore, preserving modality-specific information while exploiting complementary relationships between modalities is an important direction for robust multimodal anomaly detection.

## 3. Methods

**Figure 1:**
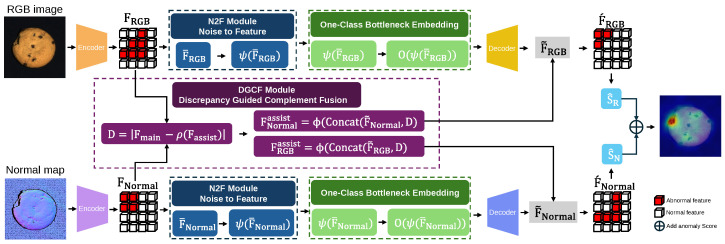
The overall framework of the proposed method.

The proposed method aims to improve unsupervised multimodal anomaly detection by effectively exploiting complementary information from different modalities while alleviating the limitations of reconstruction-based anomaly detection. The overall framework of the proposed method is illustrated in Figure 1. The framework consists of two modality-specific encoder–decoder branches, where RGB and normal inputs are processed independently to extract modality-specific feature representations. In each branch, the encoder extracts multi-scale features, and OCBE compresses these features into compact normal representations before they are passed to the decoder. This bottleneck structure helps suppress unnecessary information and guides the decoder to reconstruct normal feature representations. Anomaly scores are then calculated by measuring the discrepancy between the encoder features and the reconstructed features.

During feature reconstruction, we introduce two modules, DGCF and N2F, to enhance multimodal feature utilization and improve reconstruction robustness. DGCF exploits the discrepancy between modalities to selectively extract complementary information from the auxiliary modality. Unlike direct summation or concatenation of heterogeneous features, this discrepancy-guided design reduces anomaly smoothing by preventing modality-specific anomaly cues from being weakened during fusion. N2F further improves the reconstruction process by injecting Gaussian noise into the feature space, which mitigates over-reconstruction and encourages the decoder to learn robust normal representations rather than simply copying the input features. Finally, anomaly maps from each modality are normalized and combined to produce the final anomaly detection result.

### 3.1. Discrepancy-Guided Complementary Fusion

In multimodal anomaly detection, directly combining features from different modalities can weaken anomaly-related responses. Since each modality contains different types of information, directly injecting auxiliary features may cause interference between modalities and lead to anomaly smoothing. This phenomenon can blur anomalous regions in the feature space and reduce the discriminability between normal and abnormal areas. To address this issue, we propose the DGCF module, which selectively extracts complementary information based on inter-modal discrepancy.

Let $F_{\mathrm{main}}$ and $F_{\mathrm{assist}}$ denote the main feature and the auxiliary feature, respectively. Here, the main feature refers to the modality currently being reconstructed, while the auxiliary feature refers to the other modality that provides complementary information. Although the proposed method uses multi-scale feature representations, the scale index is omitted for simplicity. Before computing the discrepancy map, the auxiliary feature is passed through a scale-wise convolutional projection block composed of one 3×3 convolutional layer followed by two 1×1 convolutional layers, where each convolutional layer is followed by Batch Normalization and ReLU activation. The inter-modal discrepancy map *D* is then computed by measuring the absolute difference between the main feature and the projected auxiliaryfeature:

(1) $$D=\left|F_{\mathrm{main}}-\rho \left(F_{\mathrm{assist}}\right)\right|.$$

where ρ(·) denotes the scale-wise convolutional projection block. For each reconstruction direction, the discrepancy map is computed with respect to the current main modality and its auxiliary modality. Specifically, in RGB reconstruction, the RGB feature is treated as $F_{\mathrm{main}}$ and the normal feature is treated as $F_{\mathrm{assist}}$. For normal reconstruction, this relationship is reversed.

The discrepancy map explicitly captures the difference between the two modalities and provides guidance for extracting complementary information. Using the reconstructed decoder features ${\tilde{F}}_{\mathrm{RGB}}$ and ${\tilde{F}}_{\mathrm{Normal}}$, DGCF generates auxiliary features by combining each reconstructed feature with the discrepancy map:

(2) $$F_{\mathrm{Normal}}^{\mathrm{assist}}=\phi \left(\mathrm{Concat}\left({\tilde{F}}_{\mathrm{Normal}},D\right)\right).$$

(3) $$F_{\mathrm{RGB}}^{\mathrm{assist}}=\phi \left(\mathrm{Concat}\left({\tilde{F}}_{\mathrm{RGB}},D\right)\right).$$

Here, ϕ(·) denotes a mapping function composed of three convolutional layers. For an input feature with *C* channels, ϕ(·) first applies a 3×3 convolution that preserves the number of channels. It then applies two 1×1 convolutions, where the first convolution expands the number of channels to 2C and the second convolution restores it to *C*. Each convolutional layer is followed by Batch Normalization and ReLU activation. Unlike direct feature fusion, DGCF does not simply combine heterogeneous modality features. Instead, it uses the discrepancy map to guide the extraction of complementary information from the reconstructed feature space. The generated auxiliary features are injected into the reconstructed features of the opposite modality. To ensure stable training, the injection strength is controlled by a learnable scaling parameter α. The parameter is constrained using a hyperbolic tangent function:

(4) $$\alpha =0.1\cdot \tanh(\tilde{\alpha }).$$

where $\tilde{\alpha }$ is a learnable parameter. This constraint prevents the auxiliary features from dominating the reconstructed features during training. The final output features of the RGB and normal modalities are defined as follows:

(5) $${\overset{\prime }{F}}_{\mathrm{RGB}}={\tilde{F}}_{\mathrm{RGB}}+\alpha F_{\mathrm{Normal}}^{\mathrm{assist}}.$$

(6) $${\overset{\prime }{F}}_{\mathrm{Normal}}={\tilde{F}}_{\mathrm{Normal}}+\alpha F_{\mathrm{RGB}}^{\mathrm{assist}}.$$

Through this process, DGCF selectively incorporates discrepancy-aware complementary information into each modality. As a result, the proposed module alleviates anomaly smoothing caused by direct multimodal feature fusion and enhances anomaly-related responses in the reconstructed feature space.

### 3.2. Noise to Feature

Reconstruction-based anomaly detection methods commonly assume that a model trained only on normal samples cannot accurately reconstruct abnormal regions. However, when the decoder has excessive reconstruction capacity, it may reconstruct abnormal regions as well as normal regions. This over-reconstruction problem reduces the difference between input and reconstructed features, thereby degrading anomaly detection performance. To alleviate this issue, we propose the N2F module. The key idea of N2F is to inject Gaussian noise into the encoder feature space before reconstruction. By perturbing the input features, the decoder is encouraged to learn robust normal patterns rather than simply copying the input features. For the encoder features $F_{\mathrm{RGB}}$ and $F_{\mathrm{Normal}}$, Gaussian noise ϵ is added as follows:

(7) $${\bar{F}}_{\mathrm{RGB}}=F_{\mathrm{RGB}}+\epsilon .$$

(8) $${\bar{F}}_{\mathrm{Normal}}=F_{\mathrm{Normal}}+\epsilon .$$

The noise term follows $\epsilon ∼N(0,\sigma^{2})$, where σ controls the noise intensity. To stabilize the perturbed features, a feature refinement function ψ(·) is applied after noise injection:

(9) $$\psi \left({\bar{F}}_{\mathrm{RGB}}\right).$$

(10) $$\psi \left({\bar{F}}_{\mathrm{Normal}}\right).$$

The function ψ(·) is implemented as a residual refinement block applied to ${\bar{F}}_{\mathrm{RGB}}$ and ${\bar{F}}_{\mathrm{Normal}}$. It consists of a 3×3 convolution, Instance Normalization, LeakyReLU activation, and another 3×3 convolution. Both convolutional layers preserve the channel dimensionality. The entire block generates a residual correction, which is added to ${\bar{F}}_{\mathrm{RGB}}$ and ${\bar{F}}_{\mathrm{Normal}}$ to obtain the refined representations $\psi \left({\bar{F}}_{\mathrm{RGB}}\right)$ and $\psi \left({\bar{F}}_{\mathrm{Normal}}\right)$, respectively. After this refinement process, the refined representations are passed through the OCBE to produce bottleneck representations, denoted as $O\left(\psi \left({\bar{F}}_{\mathrm{RGB}}\right)\right)$ and $O\left(\psi \left({\bar{F}}_{\mathrm{Normal}}\right)\right)$, respectively. The resulting OCBE outputs are then used as the inputs to the decoder. By introducing feature-level perturbation, N2F discourages the decoder from directly reproducing the input features and helps preserve the discrepancy between normal and abnormal feature representations.

### 3.3. Anomaly Scoring

During inference, anomaly scores are computed by measuring the difference between the encoder features and the final reconstructed features enhanced by DGCF. Specifically, cosine distance is used to calculate the anomaly score for each modality. The anomaly scores for the RGB and normal modalities are defined as follows:

(11) $$S_{R}=1-\mathrm{Sim}\left(F_{\mathrm{RGB}},{\overset{\prime }{F}}_{\mathrm{RGB}}\right).$$

(12) $$S_{N}=1-\mathrm{Sim}\left(F_{\mathrm{Normal}},{\overset{\prime }{F}}_{\mathrm{Normal}}\right).$$

Here, Sim(·) denotes cosine similarity. A higher score indicates a larger discrepancy between the encoder feature and the final reconstructed feature, which suggests a higher likelihood of anomaly. The anomaly maps obtained from the feature representations are upsampled to the input image resolution and aggregated. Before combining the two modalities, the RGB and normal anomaly maps are normalized separately using modality-wise statistics computed from the validation set. Specifically, z-score normalization is applied as follows:

(13) $${\hat{S}}_{R}=\frac{S_{R}-\mu_{R}}{\sigma_{R}},\;{\hat{S}}_{N}=\frac{S_{N}-\mu_{N}}{\sigma_{N}}.$$

where $\mu_{R}$ and $\sigma_{R}$ denote the mean and standard deviation of the RGB anomaly maps, and $\mu_{N}$ and $\sigma_{N}$ denote those of the normal anomaly maps, respectively. These statistics are computed on the validation set and fixed during testing. The final anomaly map is computed by combining the normalized anomaly maps from the RGB and normal modalities:

(14) $$S={\hat{S}}_{R}+{\hat{S}}_{N}.$$

This modality-wise normalization prevents one modality from dominating the final anomaly map due to differences in score scale or distribution. The final anomaly map *S* is used for pixel-level anomaly localization, and the image-level anomaly score is obtained by taking the maximum value of the anomaly map.

## 4. Experiments

### 4.1. Datasets

The proposed method was evaluated on two multimodal industrial anomaly detection datasets: MVTec 3D-AD [22] and Eyecandies [23]. MVTec 3D-AD is a real-world industrial anomaly detection dataset that provides RGB images and depth information. It contains various object categories and defect types, including practical noise commonly observed in industrial environments. Therefore, this dataset is suitable for evaluating the robustness of anomaly detection methods under realistic conditions.

Eyecandies is a generated industrial anomaly detection dataset that provides RGB, depth, and normal information. Compared with MVTec 3D-AD, Eyecandies contains relatively low-noise and structurally well-organized data. Thus, it is useful for analyzing the effectiveness of multimodal anomaly detection methods in a more controlled environment.

In this study, the depth information in MVTec 3D-AD was converted into normal maps and used together with RGB images. For Eyecandies, RGB and normal modalities were used in the experiments.

### 4.2. Evaluation Metrics

To quantitatively evaluate anomaly detection performance, three evaluation metrics were used: image-level AUROC (I-AUROC), pixel-level AUROC (P-AUROC), and AUPRO. I-AUROC evaluates the ability to distinguish normal and anomalous samples at the image level, while P-AUROC measures the localization performance at the pixel level. AUPRO evaluates the overlap between the predicted anomaly regions and the ground-truth anomaly regions. In this study, AUPRO was calculated within the false positive rate (FPR) range of up to 30%.

### 4.3. Experimental Setup

All experiments were conducted on a workstation equipped with an NVIDIA RTX 3090 GPU. The same hyperparameter settings were used for both MVTec 3D-AD and Eyecandies. The input images were resized to 256×256 pixels for both training and evaluation. WideResNet50 was used as the backbone network for both the encoder and decoder, and feature maps from layer 1, layer 2, and layer 3 were used. During inference, anomaly maps were computed at each of the three feature levels, upsampled to the input resolution, and summed to obtain the modality-specific anomaly map. The model was trained for 200 epochs with a batch size of 16, and the initial learning rate was set to 0.005. To ensure reproducibility, the random seed was fixed at 42 for all experiments.

### 4.4. Quantitative Results

The proposed method was compared with existing unsupervised multimodal anomaly detection methods, including M3DM [21], AST [5], and CFM [24]. The quantitative results on MVTec 3D-AD are presented in Table 1. The proposed method achieved an I-AUROC of 97.3%, a P-AUROC of 99.6%, and an AUPRO of 97.6%. These results show that the proposed method provides stable anomaly detection performance at both the image and pixel levels. The performance improvement can be attributed to the complementary roles of DGCF and N2F. DGCF utilizes the discrepancy between RGB and normal features rather than directly combining multimodal features. This design helps reduce anomaly smoothing, where anomaly-related responses from one modality may be weakened during feature fusion. By emphasizing discrepancy-based complementary information, DGCF preserves modality-specific anomaly cues and improves the discriminability of abnormal regions. In addition, N2F injects noise into the feature space during reconstruction, which prevents the decoder from simply copying the input features. As a result, the model is encouraged to reconstruct robust normal representations, while anomalous features remain difficult to reconstruct. This contributes to maintaining a clear difference between the input and reconstructed features, which is important for reconstruction-based anomaly detection.

The quantitative results on Eyecandies are shown in Table 2. The proposed method achieved average scores of 94.8% I-AUROC, 98.6% P-AUROC, and 93.4% AUPRO. These results indicate that the proposed method effectively utilizes complementary information from RGB and normal modalities. In particular, the performance improvement suggests that discrepancy-guided feature fusion can help preserve anomaly-related responses during multimodal feature reconstruction. Furthermore, the consistent pixel-level performance demonstrates that N2F helps suppress over-reconstruction and supports more reliable anomaly localization.

Overall, the results on both datasets demonstrate the effectiveness of the proposed method in unsupervised multimodal industrial anomaly detection. DGCF improves multimodal feature fusion by selectively using discrepancy-guided complementary information, while N2F enhances the reconstruction process by reducing over-reconstruction. The proposed method achieves robust performance not only on the real-world MVTec 3D-AD dataset but also on the more controlled Eyecandies dataset, indicating its applicability to different multimodal anomaly detection environments.

### 4.5. Qualitative Results

**Figure 2:**
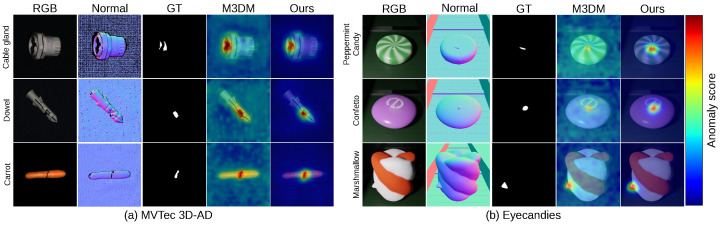
Qualitative anomaly localization examples on the MVTec 3D-AD and Eyecandies datasets. The figure shows RGB images, normal maps, ground truth and predicted anomaly maps, where warmer colors indicate higher anomaly scores.

The qualitative results are shown in Figure 2. Existing methods tend to generate blurred anomaly maps or activate surrounding background regions together with defective areas. This indicates that anomaly-related responses may be weakened or dispersed during the detection process, especially when multimodal features are not effectively combined. In contrast, the proposed method produces anomaly maps that are more concentrated on actual defective regions. This result can be attributed to the proposed DGCF module, which utilizes discrepancy-guided complementary information rather than directly combining features from different modalities. By focusing on the differences between RGB and normal features, DGCF helps preserve modality-specific anomaly cues and reduces the dispersion of anomaly responses during multimodal fusion.

Furthermore, N2F contributes to producing more clearly localized anomaly maps by reducing over-reconstruction in the decoder. Since noise is injected into the feature space during reconstruction, the decoder is encouraged to learn robust normal representations instead of simply reproducing the input features. As a result, abnormal regions remain more distinguishable from reconstructed normal features, leading to more precise anomaly localization. These qualitative observations are consistent with the quantitative results and further support the effectiveness of the proposed multimodal fusion and reconstruction strategy. In particular, the concentrated anomaly responses demonstrate that DGCF and N2F help preserve anomaly-related information while suppressing unnecessary background activations.

### 4.6. Ablation Study

To analyze the effectiveness of the proposed DGCF module, we first conducted an ablation study by comparing DGCF with an early fusion strategy. In this study, the early fusion baseline combines RGB and normal inputs before feature extraction. Specifically, the two inputs are merged by pixel-wise weighted summation with equal weights as follows:

(15) $$I_{\mathrm{fused}}=0.5I_{\mathrm{RGB}}+0.5I_{\mathrm{Normal}}.$$

The fused image is then used as a single input for anomaly detection, whereas DGCF maintains modality-specific feature streams and performs discrepancy-guided feature fusion during the reconstruction process. The results are summarized in Table 3. On MVTec 3D-AD, DGCF improved I-AUROC, P-AUROC, and AUPRO by 0.8%, 0.1%, and 0.3%, respectively, compared with early fusion. On Eyecandies, DGCF achieved improvements of 4.5%, 0.5%, and 1.8% in I-AUROC, P-AUROC, and AUPRO, respectively. These results show that DGCF is more effective than direct input-level fusion because it exploits discrepancy information between modalities to extract complementary information.

We also conducted an ablation study to evaluate the contribution of each proposed module. The results are presented in Table 4. On MVTec 3D-AD, DGCF significantly improved I-AUROC and acted as the main factor contributing to the overall performance gain. This result indicates that selectively incorporating complementary information based on inter-modal discrepancy can reduce anomaly smoothing and improve the separability of anomalous regions. The N2F module showed relatively limited improvement when applied alone on MVTec 3D-AD, and its additional contribution was also small when combined with DGCF. This may be because DGCF already provides sufficient discriminative capability on this dataset, making the effect of feature perturbation less pronounced. In addition, the relatively small gain of N2F may be related to the inherent characteristics of MVTec 3D-AD. Because MVTec 3D-AD is a real-world dataset acquired using physical sensors, even normal samples can contain acquisition noise, geometric irregularities, and modality-specific variations. Such inherent variability may already make exact feature reconstruction less straightforward, thereby reducing the marginal benefit of the additional feature-level perturbation introduced by N2F. On Eyecandies, the best performance was achieved when DGCF and N2F were applied together. This result suggests that the two modules work in a complementary manner. Since Eyecandies contains relatively clean and well-structured data, feature-level perturbation through N2F can more effectively regularize the reconstruction process and alleviate over-reconstruction. These results indicate that DGCF mainly contributes to reducing anomaly smoothing, while N2F further improves robustness by mitigating over-reconstruction depending on the dataset characteristics.

To further analyze the robustness of the proposed design, we conducted an additional hyperparameter sensitivity analysis on MVTec 3D-AD. Specifically, we evaluated the influence of the α scale factor in DGCF and the noise standard deviation σ in N2F. In the main experiments, the default settings are α scale = 0.10 and σ=0.05. To examine the effect of each parameter independently, one parameter is varied while the other is fixed to its default value. As shown in Table 5, the proposed method shows stable performance across all tested settings. When the α scale factor is varied from 0.05 to 0.20, the performance remains consistent, with only minor differences in I-AUROC, P-AUROC, and AUPRO. Similarly, varying σ from 0.03 to 0.07 results in nearly identical localization performance, while the image-level AUROC also remains stable. These results indicate that the proposed framework is not highly sensitive to these hyperparameters and maintains stable performance under different parameter settings.

In addition, we evaluated the computational cost of the proposed framework on MVTec 3D-AD. All measurements were conducted on a single NVIDIA RTX 3090 GPU with a batch size of 1 and an input resolution of 256×256. Each measurement was repeated five times, and the mean and standard deviation are reported for latency and FPS. As shown in Table 6, the proposed method increases the number of parameters from 369.1 M to 528.8 M and the peak VRAM usage from 1581.8 MB to 2222.9 MB compared with the baseline. The inference latency increases from 102.8±0.9 ms/image to 114.1±0.7 ms/image, while the inference speed decreases from 9.7±0.1 FPS to 8.8±0.1 FPS. These results indicate that the proposed framework introduces additional computational cost due to the DGCF and N2F modules, but still maintains a practical inference speed.

## 5. Conclusions

In this paper, we proposed the DGCF and N2F modules to simultaneously alleviate the anomaly smoothing problem in unsupervised multimodal anomaly detection and the over-reconstruction problem in reconstruction-based approaches. Existing multimodal methods based on early fusion are limited because anomaly-related information can be weakened by interference between different modalities. To address this issue, the proposed DGCF module does not directly combine auxiliary modality features. Instead, it extracts complementary information based on the discrepancy between the current modality and the auxiliary modality, and selectively injects the extracted information to reduce anomaly smoothing. In addition, the proposed N2F module injects Gaussian noise into the encoder feature space and stabilizes the perturbed features before feeding them into the decoder. This process discourages the decoder from simply copying the input features and helps suppress over-reconstruction. Experimental results on the MVTec 3D-AD and Eyecandies datasets showed that the proposed method achieved consistent quantitative and qualitative improvements compared with existing methods. These results demonstrate that the proposed approach can alleviate the limitations of simple fusion-based multimodal methods and more effectively utilize complementary information between modalities.

## Figures and Tables

**Table 1:** Quantitative results on the MVTec 3D-AD dataset. The best results are shown in **bold**, and the second-best results are underlined.

I-AUROC											
Metrics	Bagel	Cable gland	Carrot	Cookie	Dowel	Foam	Peach	Potato	Rope	Tire	Average
M3DM [21]	99.4	90.9	97.2	97.6	96.0	94.2	97.3	89.9	97.2	85.0	94.5
AST [5]	98.3	87.3	97.6	97.1	93.2	88.5	97.4	**98.1**	**100.0**	79.7	93.7
CFM [24]	98.8	87.5	**98.4**	**99.2**	99.7	92.4	96.4	94.9	97.9	**95.0**	96.0
Ours	**99.7**	**99.3**	97.8	96.3	**100.0**	**95.4**	**99.3**	94.7	99.8	91.0	**97.3**
**P-AUROC**											
Metrics	Bagel	Cable gland	Carrot	Cookie	Dowel	Foam	Peach	Potato	Rope	Tire	Average
M3DM [21]	99.5	99.3	99.7	98.5	98.5	98.4	99.6	99.4	99.7	99.6	99.2
AST [5]	-	-	-	-	-	-	-	-	-	-	97.6
CFM [24]	**99.7**	99.2	**99.9**	97.2	98.7	99.3	**99.8**	**99.9**	**99.8**	**99.8**	99.3
Ours	99.2	**99.6**	99.7	**99.3**	**99.7**	**99.5**	99.7	99.7	99.5	99.6	**99.6**
**AUPRO**											
Metrics	Bagel	Cable gland	Carrot	Cookie	Dowel	Foam	Peach	Potato	Rope	Tire	Average
M3DM [21]	97.0	97.1	97.9	95.0	94.1	93.2	97.7	97.1	97.1	97.5	96.4
AST [5]	97.0	94.7	98.1	93.9	91.3	90.6	97.9	98.2	88.9	94.0	94.4
CFM [24]	**98.0**	96.6	**98.2**	94.7	95.9	96.7	**98.2**	**98.3**	**97.6**	**98.2**	97.2
Ours	97.0	**97.9**	98.1	**97.2**	**98.0**	**97.5**	97.9	98.1	96.9	97.6	**97.6**

**Table 2:** Quantitative results on the Eyecandies dataset. The best results are shown in **bold**, and the second-best results are underlined.

I-AUROC											
Metrics	C. Cane	C. Cookie	C. Praline	Confetto	G. Bear	H. Truffle	L. Sandwich	Lollipop	Marshmallow	P. Candy	Average
M3DM [21]	62.4	95.8	**95.8**	**100.0**	88.6	75.8	94.9	83.6	**100.0**	**100.0**	89.7
AST [5]	57.4	74.7	74.7	88.9	59.6	61.7	81.6	84.1	98.7	98.7	78.0
CFM [24]	68.0	93.1	95.2	88.0	86.5	78.2	91.7	84.0	99.8	96.2	88.1
Ours	**89.6**	**99.7**	94.2	97.6	**91.3**	**82.6**	**97.4**	**96.3**	**100.0**	98.7	**94.8**
**P-AUROC**											
Metrics	C. Cane	C. Cookie	C. Praline	Confetto	G. Bear	H. Truffle	L. Sandwich	Lollipop	Marshmallow	P. Candy	Average
M3DM [21]	97.4	98.7	96.2	**99.8**	**96.6**	94.1	97.1	98.4	99.6	97.7	97.7
AST [5]	76.3	96.0	91.1	96.9	78.8	83.7	91.8	92.4	98.3	96.8	90.2
CFM [24]	98.3	98.2	96.4	98.9	94.9	94.6	96.9	98.0	99.5	98.7	97.4
Ours	**99.3**	**98.8**	**98.9**	99.4	96.3	**95.6**	**99.6**	**98.9**	**99.7**	**99.3**	**98.6**
**AUPRO**											
Metrics	C. Cane	C. Cookie	C. Praline	Confetto	G. Bear	H. Truffle	L. Sandwich	Lollipop	Marshmallow	P. Candy	Average
M3DM [21]	90.6	92.3	80.3	**98.3**	85.5	68.8	88.0	90.6	96.6	95.5	88.2
AST [5]	51.4	83.5	71.4	90.5	58.7	59.0	73.6	76.9	91.8	87.8	74.4
CFM [24]	94.2	90.2	83.1	96.5	87.5	**76.2**	79.1	91.3	93.9	94.9	88.7
Ours	**96.1**	**92.9**	**94.6**	96.6	**93.2**	75.8	**97.0**	**93.5**	**97.9**	**96.5**	**93.4**

**Table 3:** Comparison of feature fusion strategies.

Method	MVTec 3D-AD			Eyecandies		
	I-AUROC	P-AUROC	AUPRO	I-AUROC	P-AUROC	AUPRO
Early fusion	96.5	99.5	97.3	90.3	98.1	91.6
DGCF	97.3	99.6	97.6	94.8	98.6	93.4

**Table 4:** Ablation study on the proposed modules.

DGCF	N2F	MVTec 3D-AD			Eyecandies		
		I-AUROC	P-AUROC	AUPRO	I-AUROC	P-AUROC	AUPRO
		95.8	99.6	97.7	92.8	98.3	93.2
✓		97.3	99.6	97.6	94.2	98.5	93.4
	✓	96.3	99.6	97.7	92.4	98.4	93.4
✓	✓	97.3	99.6	97.6	94.8	98.6	93.4

**Table 5:** Sensitivity analysis of the DGCF α scale factor and N2F noise standard deviation σ on MVTec 3D-AD.

Module	Tested Parameter	Value	Fixed Parameter	I-AUROC	P-AUROC	AUPRO
DGCF	α scale factor	0.05	σ=0.05	97.47	99.58	97.70
		0.10		97.34	99.56	97.63
		0.20		97.42	99.54	97.58
N2F	Noise standard deviation σ	0.03	α scale = 0.10	97.29	99.56	97.63
		0.05		97.34	99.56	97.63
		0.07		97.22	99.56	97.63

**Table 6:** Computational efficiency comparison between the baseline and the proposed method on MVTec 3D-AD. Measurements were conducted with a batch size of 1 and an input resolution of 256×256 on a single NVIDIA RTX 3090 GPU. Latency and FPS are reported as the mean ± standard deviation over five repeated measurements.

Method	Params (M)	Peak VRAM (MB)	Latency (ms/img)	FPS
Baseline	369.1	1581.8	102.8±0.9	9.7±0.1
Ours	528.8	2222.9	114.1±0.7	8.8±0.1

## Data Availability

The data presented in this study are available on request from the corresponding author.
